# New classification of natural breeding habitats for Neotropical anophelines in the Yanomami Indian Reserve, Amazon Region, Brazil and a new larval sampling methodology

**DOI:** 10.1590/0074-02760150168

**Published:** 2015-09

**Authors:** Jordi Sánchez-Ribas, Joseli Oliveira-Ferreira, Maria Goreti Rosa-Freitas, Lluís Trilla, Teresa Fernandes Silva-do-Nascimento

**Affiliations:** 1 Fundação Oswaldo Cruz, Instituto Oswaldo Cruz, Laboratório de Imunoparasitologia, Rio de Janeiro, RJ, Brasil; 2 Fundação Oswaldo Cruz, Instituto Oswaldo Cruz, Laboratório de Mosquitos Transmissores de Hematozoários, Rio de Janeiro, RJ, Brasil; 3 Institut de Recerca en Energia de Catalunya, Barcelona, Spain

**Keywords:** Anopheles larvae, lakes associated with rivers, effective breeding area, Yanomami

## Abstract

Here we present the first in a series of articles about the ecology of immature stages of anophelines in the Brazilian Yanomami area. We propose a new larval habitat classification and a new larval sampling methodology. We also report some preliminary results illustrating the applicability of the methodology based on data collected in the Brazilian Amazon rainforest in a longitudinal study of two remote Yanomami communities, Parafuri and Toototobi. In these areas, we mapped and classified 112 natural breeding habitats located in low-order river systems based on their association with river flood pulses, seasonality and exposure to sun. Our classification rendered seven types of larval habitats: lakes associated with the river, which are subdivided into oxbow lakes and nonoxbow lakes, flooded areas associated with the river, flooded areas not associated with the river, rainfall pools, small forest streams, medium forest streams and rivers. The methodology for larval sampling was based on the accurate quantification of the effective breeding area, taking into account the area of the perimeter and subtypes of microenvironments present per larval habitat type using a laser range finder and a small portable inflatable boat. The new classification and new sampling methodology proposed herein may be useful in vector control programs.

Nearly all malaria cases in Brazil are reported from the Amazon Region (99.8%), where malaria remains a public health problem (Oliveira-Ferreira et al. 2010). Between 2001-2013, an average of 392,600 malaria cases per year was recorded in Brazil. Although there was a decreasing trend in the number of malaria cases nationwide, from half a million annual cases during the 1990s to 130 thousand malaria cases currently, highly focal hotspots of malaria transmission persist, especially for *Plasmodium vivax *(de Pina-Costa et al. 2014). In 2011, approximately 10.6% of all malaria cases in the Amazon Region were registered in indigenous areas, with higher incidences in certain states, such as Amazonas (AM), where 49% of the cases were reported in indigenous villages (MS/ SVS 2013). Despite this critical malaria burden in indigenous areas of the Amazon Region, few studies have reported in detail how malaria transmission is sustained in these remote areas or the ecology of anopheline larvae in such settings.

In Brazil, *Anopheles darlingi*, *Anopheles albitarsis s.l.* and *Anopheles aquasalis *are considered the main malaria vectors in different areas of the Amazon Region. *Anopheles nuneztovari s.l.* and *Anopheles triannulatus s.l.* also play a role in malaria transmission as secondary or occasional vectors in some areas (Deane et al. 1948, 1986, Rosa-Freitas et al. 1998, Pimenta et al. 2015). Studies of anophelines in undisturbed and remote indigenous areas of the Amazon rainforest are difficult to perform due to their location and restricted access to these areas, which require the use of light aircrafts, boats and/or many walking hours to reach them. This is the case of the Yanomami areas studied herein. The Yanomami are a numerous, semi-isolated indigenous group living in the northern part of the Brazilian Amazon and southern Venezuela (Pithan et al. 1991).

For the ecological study of anopheline larvae, it is crucial to correctly identify and classify the main breeding sites in the area and thus to monitor their dynamics over time.

Lakes along the floodplains of Amazonian white-water rivers, known as*várzeas*, have been classified by Junk et al. (2012) into four types: scroll lakes (i.e., elongated lakes that are narrow and mostly covered by aquatic macrophytes), oxbow lakes (OX) (originating from abandoned river meanders with a horseshoe shape), depression lakes (located in floodplain depressions) and upland ria-lakes (i.e., dendritic lakes formed in drowned valleys of tributary rivers). OX may be connected in various degrees to the river and this connection greatly influences the seasonal water quality and volume as well as the macrophyte and invertebrate fauna composition (Mormul et al. 2013). According to the Strahler (1957) stream order classification, rivers are categorised based on a hierarchy of tributaries. The flood pulse, defined as the pulsing events of a river discharge, has been proposed as the principal driving force of biota dynamics in river-floodplain systems (Junk et al. 1989). Therefore, in the Amazonian rivers and their tributaries, characteristics such as river order level and relation with flood pulsing, river width and watercourse sinuosity will determine the abundance and characteristics of the natural breeding habitats, the distribution of anopheline species and their densities, and will thus influence local malaria patterns of transmission.

In general, studies of anopheline larvae present difficulties related to the great diversity of larval habitats used by anopheline females, some of which are difficult to access (Rejmankova et al. 2013). Larval habitats of the major African malaria vectors within the *Anopheles gambiae*species complex, for example, are typically small, sunlit, temporary and with turbid waters. These larval habitats are typically created as a result of human or animal activities and they may be difficult to locate, especially during the wet season (Mutuku et al. 2006). However, within the Amazon Basin, some breeding habitats may be relatively large (Deane et al. 1948) and may present microhabitats. These large breeding habitats require specific tools to cover all potential breeding microhabitats within their water body in order to avoid biased sampling results.

In this paper, we propose a comprehensive classification of larval habitats and present a new methodology to investigate the natural breeding habitats of anopheline larvae in areas of low-order rivers in the Brazilian Amazon. We also included some preliminary results regarding the application of a new larval field methodology.

## MATERIALS AND METHODS

**Fig. 1:**
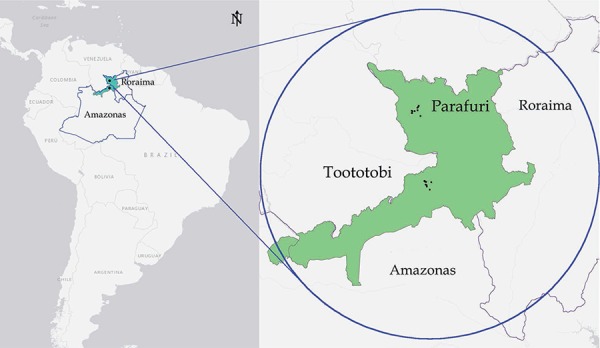
: Brazil, the states of Roraima and Amazonas, the Yanomami indigenous communities, Parafuri and Toototobi.

*Study area *- We conducted a longitudinal study from January 2013-July 2014 in two remote Yanomami areas of Brazil, the communities of Parafuri (3º17’1.68”N 63º51’2.16”W, 440 m) and Toototobi (1º45’54.72”N 63º37’7.68”W, 128 m), which are located in western state of Roraima (RR) and northern AM, respectively (Fig. 1). Parafuri and Toototobi are communities composed of six and seven villages, respectively, and each village may comprise one big, circular, central open hut (called a *shabono*) or by smaller and separated huts. We selected four villages to study in each community based on malaria incidence and for logistical reasons. In Parafuri and Toototobi, systematic ground surveys were conducted at two-month intervals for 19 months; during each survey we performed anopheline collections for 15 consecutive days. The Yanomami are the major indigenous ethnic group living semi-isolated in an area of 192,000 km^2^, spanning the northern Brazilian Amazon and adjacent areas of the southern of Venezuela (Fig. 1) (Pithan et al. 1991). The Parafuri and Toototobi communities occupy different ecological areas, with diverse geomorphologic, hydrologic and larval habitat dynamics and with different malaria vector species compositions and abundances. Parafuri is located in a submontane tropical rainforest ecoregion. The submontane ecoregion of RR is located exclusively inside the Yanomami reserve at 350-650 m in altitude, while Toototobi, which is located 170 km south of Parafuri, lies in a lowland tropical rainforest ecoregion of Amazon with a maximum of 150 m in altitude (Rosa-Freitas et al. 2007).

*Classification of anopheline breeding habitats - *During fieldwork, we observed that there were many different types of breeding habitats for which there existed no classification that would fulfil our needs. Therefore, we classified the breeding habitats where we collected anopheline larvae based on three main environmental variables: seasonality (permanent or temporary), sun exposure (shadowed, partially or fully exposed to the sun) and association with low-order river flood pulses (larval habitats associated with large fluctuations in water river levels). All breeding habitats were georeferenced. During the dry season, ponds in small and medium sized forest streams of Toototobi were mapped and terrain data were acquired with a hand-held global positioning system (GPS) (GPSMAP 78 s, Garmin^®^, USA). We also mapped most forest streams in Toototobi, typically starting at the nearest point from each Yanomami hut, following the watercourse in both the upstream and downstream directions up to 1 km distance from the hut.

*Methodology of sampling of anopheline larvae in natural breeding habitats in the Brazilian Amazon -* In the two Yanomami communities, Parafuri and Toototobi, systematic mosquito collections were conducted in each of the eight villages (4 in Toototobi and 4 in Parafuri) and individuals were asked to note the location of known water bodies within a 1 km range of the village. An initial sketch was made of the location of each water body with respect to the village. Afterwards, all of the water bodies were located with the help of a Yanomami guide and mapped by GPS; almost all breeding habitats were surveyed and anopheline larvae were sampled.

We carried out a pilot study in September 2012 in both Yanomami areas with the following objectives: (i) to map all breeding habitats, (ii) to establish a local sampling strategy according to time constraints for each village, (iii) to test the new sampling larvae methodology for sampling larvae and refine possible pitfalls on-site and (iv) to obtain a preliminary idea of the composition of anopheline fauna around the eight Yanomami villages.

We observed that only some microhabitats inside the breeding habitat were suitable for anopheline immature stages to thrive and calculated the effective breeding area (EBA). The EBA was defined as the area in m^2^ of a given breeding habitat that is suitable for the occurrence of immature stages of Neotropical anophelines. One EBA unit equals 1 m^2^. To calculate the EBA and sample the different breeding habitats, we used a laser measurement tool (Laser Rangefinder Scout DX 1000 ARC, Bushnell^®^, USA) and a small inflatable boat (Atlantic 200, Nautika^®^, Brazil). The laser tool was able to accurately measure distances under dense canopy and under low light conditions. One limitation of this technique is that the laser tool cannot measure distances ≤ 5 m. Therefore, such short distances needed to be visually estimated, which may lead to some measurement inaccuracies. Thus, the EBA measurement technique may be less suitable for anopheline species that prefer to thrive in very small water bodies.

**Fig. 2:**
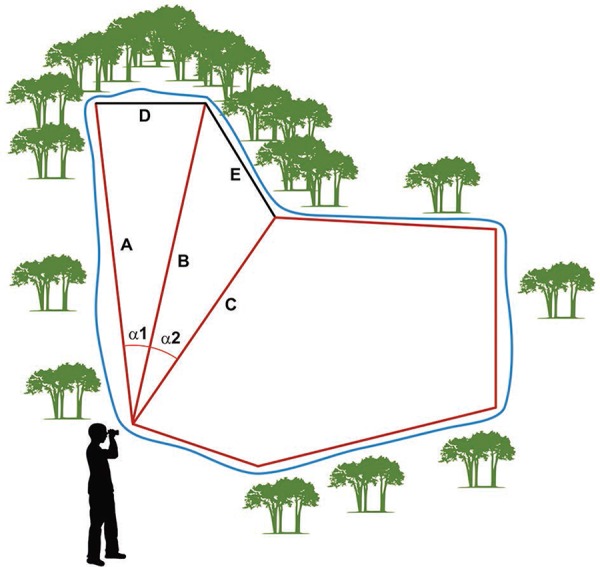
: effective breeding area measurement. Schematic approach of how was calculated difficult-to-walk-through breeding perimeter areas using a laser measurement tool. D is calculated from the measurement of A + B and angle α1, while E is calculated from B + C and angle α2.

In the case of aquatic habitats with difficult access, where a part of the perimeter could not be tracked and measured either on foot or by boat, we used a trigonometric approach to calculate the total EBA of the perimeter (pEBA) (Fig. 2). pEBA of a larval habitat is the sum of all 1 m^2^ along its shoreline. Basically, two distances on each extreme of the segment were calculated and we subsequently estimated the angle between both measurements with a half circle protractor marked in degrees.

The final distance of the unknown segment was calculated using equation 1.


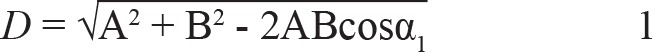


In the case of circular-shaped breeding habitats, after measuring the diameter with the laser tool, we calculated the total perimeter EBA using equation 2.


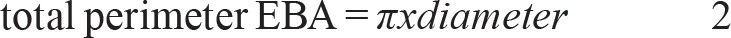


**Fig. 3:**
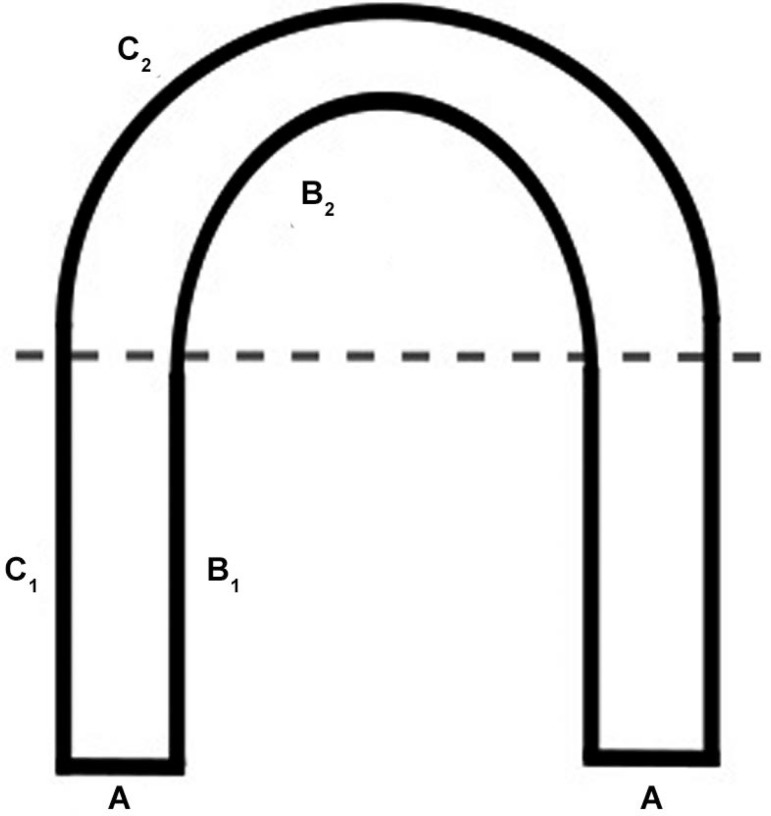
: schematic representation of the linear and curved segments of an oxbow lake used to calculate the total perimeter effective breeding area.

For large OX, we standardised accurate measurements by first measuring the perimeter only on the external side of the lake (typically the more accessible side, either by foot or by boat) and the width followed by an estimation of the internal perimeter of the water body. We used EBA measurement mainly due to time constraints and accessibility issues. EBA measurement was considered the most appropriate approach to calculate the total perimeter of a determined oxbow lake on the field within our timetable. Each oxbow lake was calculated following the same parameters in each survey. Equation 3 estimates the internal perimeters of OX.


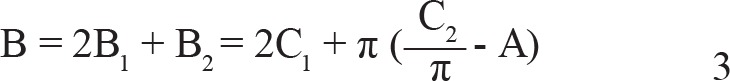


where B = total internal perimeter, B_1_ = linear segments of internal perimeter, B_2_ = curved segment of internal perimeter, C_1_ = linear segment of external perimeter, C_2_ = curved segment of external perimeter and A = width of the oxbow lake (Fig. 3).

If an oxbow lake presented a markedly irregular form, we applied the above approach but fragmented the external perimeter into fewer subunits, each consisting of two linear and one curved segments. Finally, for those OX that were nearly circular in shape, we simplified the previous equation considering that C_1 _= 0, resulting in equation 4.


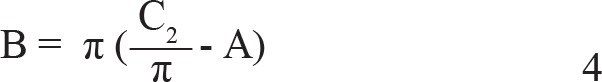


For breeding habitats where it was impossible to determine the EBA accurately, such as the massive flooded areas not associated with the river (FANAR) during the peak of the rainy season, we arbitrarily assigned a value of 500 m^2^ EBA to allow for comparisons between areas and seasons. In the case of small forest streams (SFS) and medium forest streams (MFS), microhabitats suitable for anopheline larvae are not uniformly distributed along the water courses. In order to consider these types of water bodies in the total EBA (tEBA) quantification per village, we also arbitrarily allocated 500 m^2^ EBA to those SFS and MFS that presented a substantial part of its course within a 1 km radius of the Yanomami village hut. For those forest streams that were shorter or situated distant from the village hut, we allocated 250 m^2^ EBA each. During the dry season, forest stream pools were accurately quantified if, when summed, they comprised less than 500 m^2^ EBA, otherwise a maximum of 500 m^2^ of EBA was also assigned.

Once the total pEBA was calculated per breeding site, we added 0%, 10%, 25% or 50% of additional EBA in respect to the pEBA depending on whether we found no (0%), low (10%), medium (25%) or high (50%) abundance of additional EBA other than the pEBA along the water body margins. These additional EBA subtypes could be patches of floating debris, clusters of submersed and emergent macrophytes or small terrain “islands” inside the water body that could create additional “internal” perimeter-like EBA in addition to those of the global perimeter of the water body shoreline. Adjusting for the additional EBA, we obtained the tEBA for each larval habitat. In the case of lakes associated with the river (LAR) where different subtypes of EBA were present, we quantified the EBA for each of the EBA subtypes. During our pilot study in September 2012, we detected high aggregation patterns of immature anopheline stages; some breeding sites were classified as positive if the majority of its tEBA was sampled. Therefore, we established a rule to try to sample the 50% of EBA for water bodies up to 200 m^2 ^tEBA. From the calculated tEBA, we determined the total number of dips. For example, if a larval habitat computed 110 m^2^ of tEBA, we equally distributed 55 sampling points along the breeding site. For breeding sites larger than 200 EBA units in m^2^ and for those whose size did not allow measurement, we established a maximum of 100 sampling points to cover as many possible different subtypes of EBA and water body surfaces. If different subtypes of EBA were present, we uniformly distributed sampling points in the different microhabitats (for example: 60 dippings in each EBA subtype). For sampling, we used a 350 mL, 13 cm diameter dipper (BioQuip, USA) and two dippings were conducted per EBA unit/m^2^/sampling point. The total number of larvae per stage for each dip was recorded and all immature stages were kept in small plastic tubes. The larvaes were later transferred to tubes with 80% ethanol for morphological identification in the laboratory. Sampling based on tEBA was preferred to capturing by units of time since there was different accessibility among breeding habitats, which improved collection efficiency. The relative abundance of culicines and the total number of predatory macroinvertebrates were recorded, along with different environmental variables such as association with the river flood pulses, distance to the village huts, degree of sunlight exposure, presence of macrophytes and filamentous algae, water turbidity and movement. The following physicochemical variables were also evaluated: pH and temperature (pH meter AK95, AKSO^®^, Brazil), salinity, total dissolved salts and conductivity (conductivity meter AK50, AKSO^®^) and dissolved oxygen (dissolved oxygen meter AK84, AKSO^®^). We estimated the degree of sun exposure using three categories: shadowed (0-25%), partially sun-exposed (25-50% and 50-75%) and sun-exposed (75-100%) breeding habitats. In other words, sun-exposed breeding habitats had between 75% and 100% of their tEBA exposed to the sun during sunlight hours. Shadowed breeding sites were typically under dense canopy and almost no sunlight reached their water surface during the day. During each survey, all breeding habitats were photographed and identified with a unique code and EBA subtypes that could be present were highlighted.

We conducted exploratory samplings in all of the three low-order rivers, including the Toototobi River in the Toototobi area and the Inajá and Parima Rivers in the mountainous area of Parafuri. River samplings were conducted up to 1 km down and upstream from each Yanomami village in Parafuri and Toototobi. We looked for typical river anopheline microhabitats such as riverbed sunlight pools, slow-flowing edges of the river with emergent, submersed vegetation or filamentous algae and microdams. Microdams are considered primary oviposition sites along rivers and have been defined as points of natural blocking of the river/stream current typically by a fallen stump or bamboo clumps with dense root systems that impede water flow and favour the accumulation of floating debris, allowing anopheline larvae to thrive (Manguin et al. 1996, Barros et al. 2011, Rejmankova et al. 2013). For small and MFS, we conducted our sampling in the areas in which we were most likely to find anopheline immature stages, such as slow-flowing margins with emergent vegetation, clusters of floating debris and other microhabitats of filamentous algae and submersed macrophytes in those few spots in the dense forest where sunlight had access to the water body surface.

## RESULTS

**Fig. 4:**
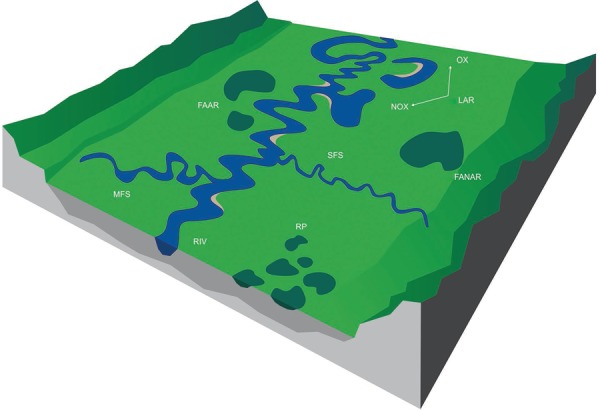
: classification of the different anophelines breeding habitats and their association with low-order rivers found in the Yanomami area, Brazilian Amazon. FAAR: flooded areas associated with the river; FANAR: flooded areas not associated with the river; LAR: lakes associated with the river; MFS: medium forest streams; NOX: nonoxbow lakes; OX: oxbow lakes; RIV: rivers; RP: rainfall pools; SFS: small forest streams.

*Classification of anopheline natural breeding habitats in low-order rivers of the Brazilian Amazon -* Breeding habitats were categorised into seven types: LAR, which are subdivided in OX and non-OX (NOX), flooded areas associated with the river (FAAR), flooded areas not associated with the river (FANAR), rainfall pools (RP), SFS, MFS and rivers (RIV) (Fig. 4).

A detailed description of the classified breeding habitats follows:

*LAR - *All anopheline breeding habitats located in the river floodplain were directly associated with river levels fluctuations. This is because breeding habitats get flooded when rivers overflow their banks; when flooding water recedes, stagnant water remains in these habitats (Drago et al. 2008). LAR may undergo variable isolation phases during rainy and dry seasons, which fluctuate in time between each flood pulse event. During the isolation phase, other sources of water, such as smaller forest streams draining into the lake, groundwater and rainfall, may maintain the lake. However, evaporation forces tend to diminish the lake water volume (Lesack & Melack 1995). According to the shape, geomorphologic origin and abundance, LAR can be further separated into OX and NOX lakes.

**Fig. 5:**
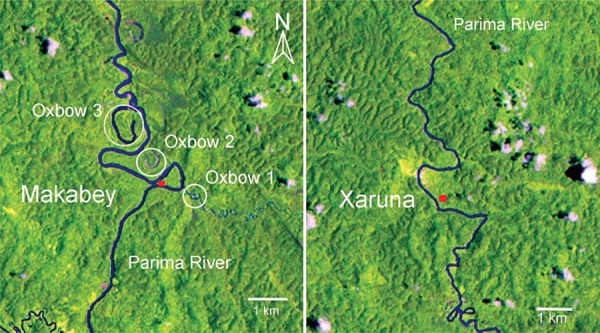
: Makabey and Xaruna villages along the Parima River in Parafuri community Yanomami indigenous area, Brazilian Amazon [LANDSAT images (MCTI 2011)]. Location of oxbow 1 and 2 of Makabey village where anopheline larvae were detected. Oxbow 1 corresponds to a U-shaped lake associated with the river that is fully connected to a small tributary of the Parima River, while oxbow 2 was an old Parima River path well disconnected from the actual river course and with very high abundance of emergent and submersed macrophytes. Only extreme flood pulses from Parima River will feed the oxbow 2. Oxbow 3 was not sampled, as it was located more than 1 km from the Yanomami huts. Within 1 km radius of Xaruna no oxbow lake was found since the Parima River is less meandering.

*OX - *OX are LAR formed by old river paths and may have a U-shaped form. U-shaped LAR were formed from the isolation of a river meander through erosion and sedimentation forces along the river edges (Esteves 1998). OX sizes vary considerably depending on the old river path that was isolated from the active river canal. The 17 OX sampled during January 2013-July 2014 were permanent even during the driest months of the year and were mostly partial or well exposed to the sun. Two LANDSAT images illustrate different segments of the Parima River that cross the Parafuri Yanomami area. Makabey village is located along a segment of the river that is more meandering and thus presented a considerable number of OX, while Xaruna village is located upstream along a river segment that did not present any U-shaped LAR near the village (Fig. 5).

*NOX lakes - *NOX lakes are non-U-shaped LAR permanent aquatic habitats and are typically partially or well exposed to the sun. NOX were generally smaller than OX. We classified small bodies of water ≤ 200 m^2^ as NOX based on water permanence and the association with river fluctuations. NOX lakes are not derived from the old river path and thus lack a clear U-shaped form. NOX lakes may be more important in regions of larger rivers where typical U-shaped LARs are less commonly found (Junk 2005). LAR are likely to play a major role in the local malaria transmission patterns, as LAR are important breeding habitats for some of the main Amazonian malaria vectors.

*FAAR -* FAAR are temporary breeding habitats where the main source of water comes from the river flood pulses. FAAR may have highly variable sizes and different levels of exposure to the sun. River flooding occurs frequently during the rainy season, though a few unpredictable flood pulses that lead to short floodplain inundations may also occur during the dry season months in low-order river systems (Junk et al. 2011). FAAR are a common type of breeding habitat found in Parafuri and Toototobi and because of their variable sun exposure characteristics, FAAR seasonally amplify a wide range of different anopheline species. As in the case of LAR, the place where the river feeds each of the identified FAAR was identified. As FAAR remain dry for many months, we did not find submersed macrophytes in these water bodies, but in cases where there was plentiful sun exposure, filamentous algae proliferated.

*FANAR - *FANAR are typically shadowed water bodies that are far and disconnected from the rivers. The origin of FANAR is related to increased rainfall during the wettest months of the year. FANAR sizes varied from vast areas in the peak of the rainy season to mostly dried spots during the driest months of the year. Most FANAR in the Yanomami area were seasonal and did not present submersed macrophytes or filamentous algae. Anopheline species that prefer fully shadowed breeding habitats are expected to predominate in FANAR.

*RP - *RP are temporary breeding habitats occurring in shallow, well-defined and typically small depressions in the forest soil that depend on rainfall to be filled. RP presented some similarities with FANAR as they are not associated with river flood pulses and are typically shadowed. However, RP are more ephemeral breeding sites than FANAR. In the Yanomami area RP were found to be located inside the preserved forest. RP exposed to the sun were also found after deforestation due to crop growing near the Yanomami huts. Like FANAR, anopheline species that prefer shadowed breeding habitats are expected to prevail in RP.

*SFS - *SFS are forest stream breeding habitats of ≤ 5 m of width that are typically shadowed. They are present in drained forested areas near the closest river or a bigger forest stream. Many SFS interconnect with other types of breeding habitats. For example, a distant FANAR may be connected through an SFS to an FAAR that receives water directly from the river flood pulses. Most SFS dry out towards the middle of the dry season. SFS tend to gradually change into ponds earlier during the dry season, potentially increasing anopheline densities of certain species during this period.

*MFS - *MFS are forest stream breeding habitats of 5-10 m in width that are almost always shadowed. They are present in drained forested areas near the closest river. In the submontane rainforest area of Parafuri, most forest streams, independent of their size, had a current flow, even during the driest months of the year. MFS form ponds more frequently through the mid-end of the dry season. Depending on the water flow and the amount of ponds formed by MFS, different anopheline species will be favoured.

**Fig. 6:**
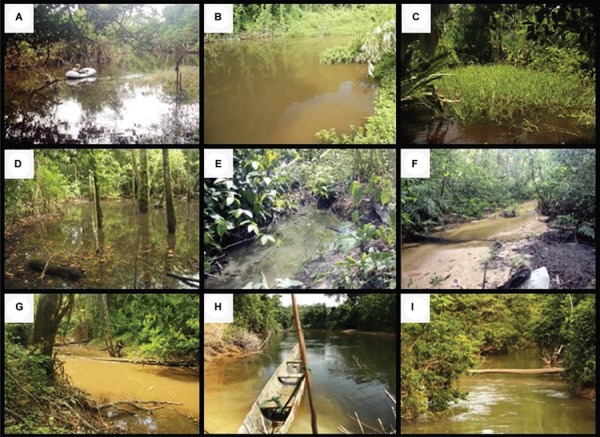
: natural anopheline breeding habitats in the Brazilian Amazon. A: U-shaped lakes associated with the river (LAR) or oxbow lake. Many*Anopheles darlingi *larvae were collected from submersed macrophytes-effective breeding area (EBA) using the small inflatable boat, a spot otherwise inaccessible on foot near Komomassipe village, Parafuri; B: non-U-shaped LAR with circular shape. As many oxbow lakes, they are permanent and well exposed to the sun; C: flooded area associated with the river, shallow and temporal breeding habitat. In this case, well exposed to the sun and with abundant additional emergent vegetation-EBA. This breeding site would correspond to the type that we would increase by 25% the EBA of the perimeter to obtain the total EBA. *Anopheles mattogrossensis, Anopheles triannulatus s.s.* and *Anopheles oswaldoi s.l.* were retrieved in high numbers from this site; D: flooded area not associated with the river, fully shadowed breeding site. A potential larval habitat for *An. oswaldoi s.l.*, *Anopheles intermedius/Anopheles guarao-*like and *Anopheles costai-*like, Apiahik village, Toototobi; E: rainfall pools, very ephemeral larval habitat, these ones were exposed to sun after forest clearance for crop cultivation and yielded many *An. darlingi* larvae, Komomassipe area; F, G: example of small (< 5 m wide) and medium (5-10 m wide) forest streams, respectively; H, I: medium-sized Inaja River in the hilly Parafuri area and Toototobi River, respectively. No *Anopheles* larvae were retrieved from these low-order rivers.

*RIV - *All rivers located in the Yanomami study area can be classified as low-order rivers (Junk et al. 2011). We further subdivided low-order rivers into small (5-15 m width), medium (15-100 m width) and large rivers (≥ 100 m width). Sizes and widths of LAR, especially for the OX, follow RIV sizes. Characteristics of large Amazon rivers differ from smaller rivers, which affects the dynamics of potential associated natural breeding sites for *Anopheles*. We present an example of each type of breeding site surveyed in Fig. 6.

*Anopheline natural breeding habitat classification in the Yanomami area studied -* During the 19 month longitudinal study in the Brazilian Yanomami area, 112 breeding sites were located, classified and sampled whenever water was present. The number of each breeding habitat type encountered in the two communities was compiled in the Table. We sampled 30 FANAR (26.8% of the total larval habitats), constituting the most abundant type of water body. The second most abundant larval habitat was LAR and we included in our study 27 of this type, which represented 24.1% of all larval habitats surveyed. Among the studied LAR, 17 LAR (63% of all LAR) were LAR-OX and 10 (37%) were LAR-NOX. The submontane area of Parafuri presented more LAR of both types (17 out of 27) than the lowland rainforest region of Toototobi (10 out of 27), with a remarkably higher number of NOX lakes (8 out of 10). However, in the Toototobi area, we encountered approximately double the amount of seasonal flooded areas than in Parafuri; both were associated with river flood pulses (FAAR - 11 out of 16) and were not associated with river fluctuations (FANAR - 20 out of 30). MFS prevalence was similar in both areas, but SFS were more predominant in Parafuri (11 out of 16), draining the numerous hills of the region to the closest river. The less common breeding site type was the ephemeral RP, which were only encountered on one occasion in Toototobi and twice in Parafuri.

**TABLE:** Breeding habitats types and their main characteristics in the Yanomami Indian Reserve, Brazilian Amazon Region

Breeding habitat type	Associated to flood pulse	Permanent	Sun exposure	Submerse macrophytes	Emergent vegetation	Filamentous algae	Current water
LAR-OX (n = 17)	3	3	0-3	0-3	2	0-3	0
LAR-NOX (n = 10)	3	3	0-3	0-3	2	0-3	0
FAAR (n = 16)	3	0	0-3	0	2	0-3	0
FANAR (n = 30)	0	0-1	0-1	0	0-1	0	0
RP (n = 3)	0	0	0-1	0	0-1	0-1	0
SFS (n = 16)	1	0-1	0-1	0-1	1	0-1	2
MFS (n = 15)	1	0-1	0-1	0-1	1	0-1	1
RIV (n = 5)	3	3	2	0	1	0	3

larval habitat characteristics were described as absent (0), low (1), medium (2) and high (3) [total number of breeding habitats in Parafuri and Toototobi (n)]. FAAR: flooded areas associated with the river; FANAR: flooded areas not associated with the river; LAR-NOX: lakes associated with the river-non-oxbow lakes; LAR-OX: LAR-OX-oxbow lakes; MFS: medium forest streams; RIV: rivers; RP: rainfall pools; SFS: small forest streams.

*Larval habitat characteristics -* Some larval habitats showed variable characteristics. A summary of the main features for the breeding habitats is presented in the Table. We considered the association with river flood pulses, seasonality and sun exposure as the three key variables to classify the larval habitats with a frequency scale of 0 (absent), 1 (low), 2 (medium) and 3 (high). For example, sun exposure classified as 0-3 for OX means that we encountered some LAR-OX that were fully shadowed while others were well exposed to the sun. When looking at water permanence in breeding habitats, all LAR were always classified as permanent (3). FAAR and RP always dried up for some months of the year (0) and some breeding habitats such as FANAR, MFS and SFS presented variability in this characteristic (0-1). For instance, of the 30 FANAR included in the study, only four (13.3%) had water in all the surveys and were considered permanent inland water bodies. Finally, the two types of LAR and all FAAR (3) were mainly formed because of the influence of river flood pulses, whereas FANAR and RP originated from rainfall waters (0). Forest streams, SFS and MFS experienced a variable influx of water from the river at high river water levels (1).

*Anopheline larvae collections, preliminary data - *We collected 6,295 anopheline immature stages, including species such as *An. darlingi*,*Anopheles oswaldoi s.l.*, *An. triannulatus s.s.*, *Anopheles mattogrossensis*, *Anophe-les intermedius/Anopheles guarao-*like and *Anopheles costai-*like. Anopheline larvae were more abundant in certain breeding habitats, as we retrieved 3,777 anophelines in LAR (of which 3,342 were obtained from U-shaped LAR), representing 60% of all larvae captured. Therefore, LAR and especially OX constituted a major breeding habitat for some of the anopheline species collected. For example, 1,356 (96.93%) *An. darlingi* and 133 (92.46%) *An. triannulatus s.s. *were collected in LAR.

*Larvae sampling methodology for anopheline natural breeding habitats in the Brazilian Amazon - *The use of a small, portable and light inflatable boat allowed us to conduct a stratified sampling covering most EBA subtypes of every LAR surveyed. The reduced size of the boat allowed us great flexibility to navigate the very shallow waters (as shallow as 25 cm in depth) and reach almost every corner of interest inside of a LAR. The pEBA encountered in the lake margins were sampled either on foot or by boat. Apart from enhanced accessibility, this device conferred excellent tested safety against life-threatening hazards that may inhabit LAR, such as the Amazonian electric eel (*Electrophorus electricus)*. Plastic boats confer excellent isolation from their electrical shocks. Sampling EBA distant from the LAR shoreline without properly using this device is not recommended. When a LAR was sampled for the first time when lake levels were very low or when water was semi-turbid or turbid, careful navigation was performed and internal navigation routes for each LAR and condition were traced. These approaches allowed us to reach most EBA subtypes within the lake and minimise boat punctures by submersed sticks. Two people, each in one boat, can collect simultaneously from the same LAR, increasing the security of the team. The use of regular metal boats or traditional indigenous wood canoes may be impractical in most LAR, especially those that are not fully and continuously connected to the river. Using this method, we saved time and the need for substantial human resources to move a heavy regular boat across the jungle, which in most cases would make larvae sampling impossible.

**Fig. 7:**
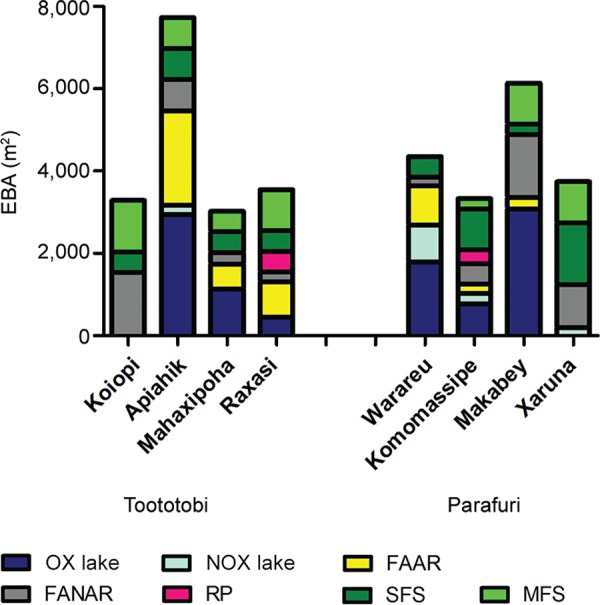
: effective breeding area (EBA) per larval habitat type in each of the Yanomami villages surveyed in September and July 2013 for Toototobi and Parafuri communities, respectively. FAAR: flooded areas associated with the river; FANAR: flooded areas not associated with the river; MFS: medium forest streams; NOX: nonoxbow lakes; OX: oxbow lakes; RP: rainfall pools; SFS: small forest streams.

*Heterogeneity of EBA availability at a microscale level *- The tEBA per breeding site type in each of the eight Yanomami villages included in the study is reported in Fig. 7. The data shown is from September 2013 fieldwork for Toototobi and from July 2013 for Parafuri, which corresponds approximately to the transitional period of the wet-to-dry season for both areas. During this period, larval habitat sizes tend to have already stabilised and most seasonal larval habitats contain water. Therefore, flooded forest areas of some larval habitats associated with the river and vast FANAR areas that are impossible to quantify during the rainy season were not encountered. We showed EBA data for Parafuri from previous field work because Parafuri anticipated the beginning of the dry season compared to Toototobi. Indeed, some aquatic sites of Parafuri during the collection of September 2013 were already dry and others presented a significantly retracted maximum EBA (especially for FANAR larval habitat type).

A marked heterogeneity of EBA availability for anopheline proliferation was encountered between both Yanomami areas and even within very close Yanomami huts of the same region. For example, while U-shaped LAR EBA was relatively abundant in communities such as Apiahik and Mahaxipoha, both in Toototobi, and Komomassipe, Warareu and Makabey located in Parafuri, U-shaped LAR EBA was not present in Xaruna (Parafuri) and Koiopi (Toototobi). However, within 1 km of the dwellings, these last two communities presented EBA available from larval habitats such as forest streams and FANAR. This variability on EBA-type occurrence will greatly influence the anopheline repertory that will predominate in the proximities of certain Yanomami communities. For example, forest-loving mosquitoes such as *An. oswaldoi s.l.*, *An. intermedius/An. guarao-*like and *An. costai-*like were predominant in Xaruna and Koiopi.

## DISCUSSION

Malaria epidemiology in relatively undisturbed areas of the Amazon Region may vary considerably between regions, depending on if they are located in areas of low or higher-order rivers and the nature of breeding sites associated to the river floodplain system. This will determine to a great extent which Neotropical malaria vectors will occur and at what densities. All rivers in our study area were low-order rivers, which are subject to short, polymodal and generally unpredictable flood pulses. These flood pulses may also occur during the drier months of the year, overflowing again the larval habitats associated with rivers during this period. This type of wetland dynamic is associated with low-order rivers between first-fifth stream order and depends to a great extent on the intensity of local rainstorms (Junk et al. 2011). Large Amazonian rivers and their floodplains are influenced by long-lasting, monomodal and predictable flood pulses (Junk et al. 2014). This flood pulse pattern is encountered along the Amazon River and its major tributaries (rivers of more than 5th stream order) (Junk et al. 2011).

There have been various classifications of larval habitats that have been used for many mosquito species (Silver 2008). Few comprehensive attempts to classify breeding sites of Neotropical anophelines have been proposed. In one proposal, larval habitats for *Anopheles albimanus* were classified in 16 clusters or habitat types and were strictly defined according to dominant botanical and limnological characteristics. The authors also ranked these 16 habitat types based on larval densities (Rejmankova et al. 1992). In their review, Sinka et al. (2010) provided a list of studies that reported the occurrence of the main Neotropical malaria vectors in some of the most commonly defined larval habitats. They were classified as large and small water bodies and as either natural or man-made. Lagoons, lakes, marshes, bogs and slow flowing rivers were considered the main large natural breeding sites, while small streams, seepage springs, pools, wells and dips in the ground were found as the most common small natural breeding sites for Neotropical anophelines. LAR would include some previously reported permanent water bodies such as lagoons and lakes.

In our study, we introduced the concept of LAR for malaria ecology, which combines the two types of larval habitats (OX and NOX lakes) that may have the most influence on malaria transmission in unaltered areas of the Amazon Basin, such as many of the indigenous areas located within low-order river systems. OX are the most frequent type of lake in the Brazilian territory, especially in the Pantanal wetlands and Amazon Basin, where a flat landscape and numerous meandering rivers favours the formation of many of these lakes (Esteves 1998). More meandering rivers tend to form more OX and thus areas with these characteristics may be more prone to become breeding habitats for *An. darlingi,* leading to malaria transmission landscapes. Different levels of hydrological connectivity propitiate colonisation by different species of macrophytes, different composition and water heaviness (Junk 2005), thus converting some LAR to a suitable breeding place for certain species of *Anopheles*. In addition, anopheline-washing episodes will be more frequent in fully connected LAR than in those that are more disconnected.

In a study carried out in the Solomon Islands, Belkin (1954) introduced the term “effective breeding surface” or “effective breeding site” to define those areas within a larval habitat suitable for anopheline breeding. Areas of open and deep standing water and water habitats with open flowing water were considered not effective breeding surfaces. In a posterior study, the “effective breeding area” in an irrigated pastureland was stratified into different microenvironments and, according to their contribution to the total breeding area, a proportional number of dips from each microenvironment were taken (Silver 2008). We used the term EBA to define those water surface areas that were productive for the different anopheline species encountered in our study areas. We refined the use of this concept by accurately quantifying the EBA of the different larval habitats with the use of a fine-scale precision and easy-to-use laser rangefinder.

With our classification, we propose a comprehensive nomenclature that attempts to simplify different denominations of natural breeding sites for low-order river systems of the Amazon. The main characteristics that we used for our classification were association to river flood pulses, seasonality and degree of sun exposure. We found that these were the best parameters to help us explain the occurrence of density hotspots of certain anopheline species and to characterise how they are dispersed during the dry and wet seasons (J Sánchez-Ribas et al., unpublished observations). Immature stage habitats were also previously classified, including water permanence (permanent, semi-permanent and temporary) and sun exposure (sunlight, partial shade and complete shade) as some of the main classification criteria (Almirón & Brewer 1996). In a larval survey in West Virginia, 15 larval types were described and mainly classified by their exposure to the sun. Habitats within the sunlit category had water surface and thus had immature mosquito stages exposed to sunlight during most daylight hours. Larval habitats located inside forests protected from direct sunlight were classified as shaded (Joy & Clay 2002).

Manguin et al. (1996) reported the use of an inflatable raft to sample microdams along the margins of the Sibun River in Belize. In our study area, the use of a smaller inflatable device was key to thoroughly sample the main breeding habitats of* An. darlingi *in our study area, the LAR. Otherwise, important anopheline microhabitats a few meters from the lake shoreline, such as submersed macrophytes EBA and floating debris exposed to the sun, would not have been accessible by foot, resulting in potential information bias regarding anopheline occurrence, larval habitat preferences and population densities.

The flight range of different anopheline species may vary, although it has been considered that the maximum distance that anophelines may flight is up to 2 km. However, it is believed that most of the flights occur at shorter distances in the 0-2 km distance range (Service 1997). According to previous reports in the literature for the flight range of Neotropical malaria vectors, we determined to collect sample from larval habitats in a range of 1 km from the Yanomami huts. This distance was considered epidemiologically relevant with a potential influence on the intra, peri and immediate extra-domiciliary transmission environments of each village. However, few studies have reported consistent data on dispersal dynamics of Neotropical anophelines. In a mark-release-recapture experiment, Charlwood and Alecrim (1989) reported most recaptures to be up to 1 km away, although a couple of *An. darlingi *were recaptured 7.2 km away from the release point. It was also noted that other Amazonian vectors such as *Anopheles strodei*,* An. triannulatus *or *An. oswaldoi*may have a more limited flight range. Achee et al. (2005) also performed a mark-release-recapture study in which they confirmed that the recapture rate for *An. darlingi* was greatest at 0 m (29%; 124/428) and decreased by 11.6% (37/318) and 5.8% (21/361) at the 400 m and 800 m recapture sites, respectively.

Disease transmission and vector ecology studies have consistently used tools such as remote sensing (RS) and Geographic Information Systems, greatly facilitating the ability to analyse relationships of vectors and diseases over time and space (Rejmankova et al. 2013). However, within the quite homogeneous landscape of undisturbed areas of the Amazon rainforest, RS may be particularly difficult to perform in areas associated with low-order river systems. For example, high-resolution satellite imagery (i.e., IKONOS images) does not exist for most of the Brazilian Yanomami area and many small FAAR and LAR, and all habitats that are under the dense canopy may be undetected on the freely available satellite images (LANDSAT images) and thus it is impossible to precisely locate and measure EBA and habitat size fluctuations. On-site accurate measurement of water bodies with this new field laser measurement approach may be the only feasible way to quantify the EBA dynamics of the different larval habitats in these remote areas of the Amazon.

*Concluding remarks -* We have proposed an easy-to-use and accurate methodology for quantifying the EBA dynamics of the main larval habitats of some key malaria vectors of the Brazilian Amazon. We believe that our classification of natural larval habitats and the new larval sampling methodology will contribute to the understanding of the influence of breeding habitat types on anopheline species-specific production and malaria transmission seasonality in low-order river systems of the Amazon. OX had enormous epidemiological relevance in our study areas, as important Amazon vectors such as *An. darlingi*, *An. triannulatus s.s.* and *An. oswaldoi s.l.* thrived in abundance within their domains. We recognise that this larvae methodology is especially suitable for some Neotropical malaria vectors, as they may breed in considerable large, spatially fixed and size variable breeding habitats. However, we also foresee applicability of the larvae methodology to accurately determine the EBA dynamics for larval habitats of other dominant malaria vectors that exploit relatively large and size-variable aquatic habitats. The larvae sampling methodology may also assist in the planning of effective control measures directed at the immature mosquito stages by precisely quantifying the tEBA (and EBA subtypes) that must be targeted during time-limited larval source management activities. Finally, with the reviewed larval habitat nomenclature, we attempted to simplify and standardise the classification of natural breeding sites in low-order river systems of the Amazon Basin.
